# Ventilation during cardiopulmonary bypass did not attenuate inflammatory response or affect postoperative outcomes

**DOI:** 10.5830/CVJA-2013-041

**Published:** 2013-08

**Authors:** Ahmet Baris Durukan, Hasan Alper Gurbuz, Halil Ibrahim Ucar, Cem Yorgancioglu, Nevriye Salman, Ertekin Utku Unal

**Affiliations:** Department of Cardiovascular Surgery, Medicana International Ankara Hospital, Ankara, Turkey; Department of Cardiovascular Surgery, Medicana International Ankara Hospital, Ankara, Turkey; Department of Cardiovascular Surgery, Medicana International Ankara Hospital, Ankara, Turkey; Department of Cardiovascular Surgery, Medicana International Ankara Hospital, Ankara, Turkey; Department of Anesthesia, Medicana International Ankara Hospital, Ankara, Turkey; Department of Cardiovascular Surgery, Ankara Yuksek Ihtisas Hospital, Ankara, Turkey

**Keywords:** cardiopulmonary bypass, respiration, artificial, lactic acid, interleukins

## Abstract

**Introduction:**

Cardiopulmonary bypass causes a series of inflammatory events that have adverse effects on the outcome. The release of cytokines, including interleukins, plays a key role in the pathophysiology of the process. Simultaneously, cessation of ventilation and pulmonary blood flow contribute to ischaemia–reperfusion injury in the lungs when reperfusion is maintained. Collapse of the lungs during cardiopulmonary bypass leads to postoperative atelectasis, which correlates with the amount of intrapulmonary shunt. Atelectasis also causes post-perfusion lung injury. In this study, we aimed to document the effects of continued low-frequency ventilation on the inflammatory response following cardiopulmonary bypass and on outcomes, particularly pulmonary function.

**Methods:**

Fifty-nine patients subjected to elective coronary bypass surgery were prospectively randomised to two groups, continuous ventilation (5 ml/kg tidal volume, 5/min frequency, zero end-expiratory pressure) and no ventilation, during cardiopulmonary bypass. Serum interleukins 6, 8 and 10 (as inflammatory markers), and serum lactate (as a marker for pulmonary injury) levels were studied, and alveolar–arterial oxygen gradient measurements were made after the induction of anaesthesia, and immediately, one and six hours after the discontinuation of cardiopulmonary bypass.

**Results:**

There were 29 patients in the non-ventilated and 30 in the continuously ventilated groups. The pre-operative demographics and intra-operative characteristics of the patients were comparable. The serum levels of interleukin 6 (IL-6) increased with time, and levels were higher in the non-ventilated group only immediately after discontinuation of cardiopulmonary bypass. IL-8 levels significantly increased only in the non-ventilated group, but the levels did not differ between the groups. Serum levels of IL-10 and lactate also increased with time, and levels of both were higher in the non-ventilated group only immediately after the discontinuation of cardiopulmonary bypass. Alveolar–arterial oxygen gradient measurements were higher in the non-ventilated group, except for six hours after the discontinuation of cardiopulmonary bypass. The intubation time, length of stay in intensive care unit and hospital, postoperative adverse events and mortality rates were not different between the groups.

**Conclusion:**

Despite higher cytokine and lactate levels and alveolar–arterial oxygen gradients in specific time periods, an attenuation in the inflammatory response following cardiopulmonary bypass due to low-frequency, low-tidal volume ventilation could not be documented. Clinical parameters concerning pulmonary and other major system functions and occurrence of postoperative adverse events were not affected by continuous ventilation.

## Abstract

Since the development of the first heart–lung machine in the 1950s, cardiopulmonary bypass (CPB) has been the only way to provide a motionless and bloodless field. During CPB, circulation is maintained by mechanical pumps and venous blood is artificially oxygenated.^1^

Cardiopulmonary bypass is a non-physiological state where blood is exposed to artificial surfaces; laminar flow is employed instead of pulsatile flow, the heart is exposed to cold cardioplegic arrest and the body temperature is lowered. These key derangements lead to a series of inflammatory events involving the endothelium, leukocytes, platelets, complement system and the coagulation cascade, with the release of various cytokines.^2^ Surgical trauma, blood product transfusion and haemodilution also participate in this inflammatory process.^3^

During CPB, pulmonary arterial circulation and alveolar ventilation are ceased and only bronchial arterial circulation supply oxygen to the lungs.^3^ After weaning from CPB, pulmonary reperfusion leads to ischaemia–reperfusion injury (I/R), with the release of oxygen free radicals and the resultant lipid peroxidation and endothelial damage.^4^ Maintaining ventilation and pulmonary flow during CPB attenuates the inflammatory response.^3^,^5^

In this study, we aimed to compare ventilation and non-ventilation regimes during CPB in patients undergoing on-pump coronary artery bypass grafting (CABG). Its influence on pulmonary injury was evaluated using lactate levels and measurement of alveolar–arterial oxygen gradients, and on postoperative cardiopulmonary functions using clinical parameters.

## Methods

A prospective, randomised study was carried out. The study was approved by the local ethics committee and written informed consent was obtained from every patient. The study followed the Declaration of Helsinki 2008 on medical protocol.

Between October 2011 and February 2012, patients subjected to elective CABG surgery were studied. Patients undergoing redo surgery, and those with pre-operative renal failure (serum creatinine > 1.3 mg/dl) or hepatic dysfunction (serum aspartate/alanine amino transferase > 40 U/l), myocardial infarction within six weeks prior to surgery, any kind of pulmonary disease, pre-operative use of steroids and ejection fraction below 30% were excluded from the study.

A total of 59 patients were prospectively randomised into two groups as follows: in the operation room, each patient was numbered chronologically by the same perfusionist. He reviewed the patient’s file prior to the induction of anaesthesia, based on a form explaining the inclusion and exclusion criteria, and the anesthesiologist was informed about the proposed regimen. Odd-numbered patients (*n* = 30) were ventilated during CPB and the even-numbered patients (*n* = 29) were not ventilated.

## Peri-operative management and anaesthesia

Pre-operative acetylsalicylic acid 100 mg/day was continued in all patients prior to the day of surgery. All patients were pre-medicated with 10 mg of oral diazepam. Anaesthesia was induced with etomidate 2 mg/kg, fentanyl 1μg/kg, vecuronium 1 mg/kg, isofluorane 1 MAC with 50% oxygen and 50% air, and remifentanyl 1μg/kg bolus, followed by a 0.5-μg/kg/min infusion. Intra-operative arterial (through the radial artery catheter) and central venous pressure (through the right internal jugular vein catheter) monitoring was done.

The CPB circuit was primed with 1 500 ml Isolyte-S® (Eczacibaşi-Baxter, Istanbul), which is a balanced electrolyte solution, and 5 000 units of heparin were added. After anticoagulation with heparin (300 U/kg), CPB was established using a roller pump with a membrane oxygenator (Dideco Compactflo Evo, Sorin group, Mirandola Modena, Italy). The average flow rate varied from 2.3 to 2.4 l/min/m^2^. Surgery was performed under mild hypothermia (33°C). Mean arterial pressure was kept between 45 and 70 mmHg. All patients were rewarmed to 37°C (nasopharayngeal temperature) before weaning from CPB. Heparin was neutralised with 1:1 protamine sulfate.

Cold (4–8°C) blood cardioplegia (1 000 ml, 25 mEq/l potassium) was administered after aortic cross clamping, and 500-ml repeat doses were given every 15 to 20 minutes (antegrade and from the venous bypass grafts; retrograde in the case of left main stenosis). Terminal warm blood cardioplegia (36–37°C) was given prior to aortic clamp release. The operating room temperature was kept at 20–21°C.

Following surgery, the patients were taken to the intensive care unit (ICU), intubated, and intravenous propofol (1–2 mg/kg/h) and morphine (0.01–0.02 mg/kg/h) were given for the maintenance of analgesia and sedation.

Atrial fibrillation (AF) was diagnosed based on an electrocardiogram. All patients were ECG monitored continuously during the ICU stay and for the first 24 hours in the ward. An ECG was immediately performed in cases of irregular pulse, palpitations or symptoms related to possible AF.

Primary outcome variables included mean time to extubation, length of stay in ICU and postoperative hospital stay, incidence of renal dysfunction (based on the finding that the peak creatinine value was ≥ 1.5 times the pre-operative value), postoperative stroke, total amount of blood loss postoperatively, postoperative exploration for haemorrhage, amount of blood and blood products used, and in-hospital mortality.

## Ventilation strategy

After intubation, before and after CPB, all patients were ventilated with F_i_O_2_ of 0.4 to 0.5, at a tidal volume of 7 ml/kg and respiratory rate of 12/min. Positive end-expiratory pressure was not used, and no requirement for FiO2 over 0.5 was required to maintain adequate oxygen saturation. The variability in ventilation parameters was to maintain a partial arterial carbon dioxide pressure between 35 and 40 mmHg.

In the non-ventilated group (NV), following establishment of CPB, ventilation was stopped (open to atmospheric pressure). In the ventilated group (V) mechanical ventilation with 0.5 F_i_O_2_ at 5 ml/kg tidal volume and 5/min respiratory rate with zero end-expiratory pressure was maintained. In the ICU, patients had volume-controlled ventilation and were extubated when their haemodynamics were stable, and neurological and respiratory functions were noted.

## Blood sampling and measurements

Peripheral arterial blood samples were collected from the radial artery catheter after the induction of anaesthesia (T_0_), and immediately after (T_1_), one (T_2_) and six hours (T_3_) after the discontinuation of CPB. The blood samples were put into citrated and EDTA anti-coagulated tubes, centrifuged, and the plasma was separated and stored at –70°C until the assay.

Plasma levels of IL-6, -8 and -10 were measured by commercially available enzyme-linked immunosorbent assays (AssayMax Human ELISA Kit, Assaypro, Missouri, USA). Plasma lactate levels were measured by blood gas analyser (ABL 700 series, Radiometer®, Brønshøj, Denmark).

Alveolar–arterial oxygen gradient (ΔA–aO2) was calculated as follows:6

ΔA–aO_2_ = P_A_O_2_ – P_a_O_2_

P_A_O_2_ = P_i_O_2_ – (P_A_CO_2_/*R*)

P_i_O_2_ = (P_B_ – PH_2_O) – F_i_O_2_

where P_A_O_2_ = alveolar oxygen tension; P_a_O_2_ = partial arterial oxygen pressure; P_i_O_2_ = partial pressure of inspired oxygen; P_A_CO_2_ = alveolar carbon dioxide tension (assumed to equal the partial arterial CO_2_ pressure due to the ease of exchange of CO_2_); *R* = respiratory quotient (assumed at 0.8); P_B_ = barometric pressure; PH_2_O = water pressure (6.2 kPa, as inspired air is fully saturated at the level of the carina); and F_i_O_2_ = fractional concentration of inspired oxygen.

## Statistical analysis

Statistical analyses were performed using SPSS software for Windows version 17.0 (Statistical Package for the Social Sciences Inc, Chicago, IL, USA). Continuous variables were expressed as mean values ± standard deviation (SD). Categorical variables were expressed as number and percentages. Demographic characteristics and outcomes of the groups were compared using independent samples *t*-test for continuous variables, and chi-square and Fisher’s exact tests for categorical variables. Statistical significance was set as *p* < 0.05.

The changes in cytokine levels, lactate and ΔA–aO_2_ within the groups were compared using the Friedman test. In cases of significant difference, Wilcoxon signed ranks test was used to define the groups that made the difference. Comparison between the groups was done using the Mann-Whitney *U*-test.

## Results

Fifty-nine patients were included in the study. There were 29 patients in the NV group and 30 in the V group. The mean ages of the patients were 62.48 ± 6.42 in the NV group and 59.40 ± 11.10 in the V group (*p* = 0.19). The pre-operative demographic data and intra-operative characteristics of the patients are given in Table 1. The groups were comparable.

**Table 1:** Pre-Operative Demographic Characteristics Of Patients

*Variable*	*NV group (n = 29) mean ± SD*	*V group (n = 30) mean ± SD*	p-value*
Age	62.48 ± 6.42	59.40 ± 11.10	0.19
BMI (kg/m^2^)	32.51 ± 12.22	29.17 ± 3.44	0.15
LVEF (%)	52.48 ± 11.77	52.57 ± 10.14	0.97
Cross-clamp time (min)	54.66 ± 13.97	54.67 ± 13.11	0.99
CPB time (min)	83.41 ± 22.72	80.83 ± 22.23	0.66
No of grafts	3.21 ± 0.77	3.03 ± 0.85	0.41
	n (%)	n (%)	p-value**
Male	23 (79.3)	26 (86.7)	0.45
Current/ex-smoker	22 (75.9)	22 (73.3)	0.82
Diabetes mellitus	16 (55.2)	12 (40.0)	0.24
Insulin dependent_a_	7 (46.7)	8 (58.3)	0.22
Hypertension	22 (75.9)	16 (53.3)	0.07
Dyslipidaemia	23 (79.3)	21 (70)	0.41
Statin therapy_b_	14 (60.9)	16 (76.2)	0.27
Pre-operative β-blocker use	17 (58.6)	12 (40.0)	0.15
Peripheral arterial disease_c_	2 (6.9)	0	0.23***
Stroke	2 (6.9)	1(3.3)	0.49***
Carotid disease_d_	0	2 (6.7)	0.61***

SD: standard deviation, NV: non-ventilated, V: ventilated, BMI: body mass index, LVEF: left ventricular ejection fraction, CPB: cardiopulmonary bypass. *Independent samples *t*-test, **Chi-square test, ***Fisher’s exact test. ^a^The percentage values were calculated in diabetic patients. ^b^The percentage values were calculated in dyslipidaemic patients. ^c^History of therapeutic vascular intervention, history of claudication, aniography/non-invasive proven peripheral arterial disease. ^d^History of carotid intervention or angiographic/non-invasive proven > 40% stenosis of either carotid artery.

**Fig. 1.**
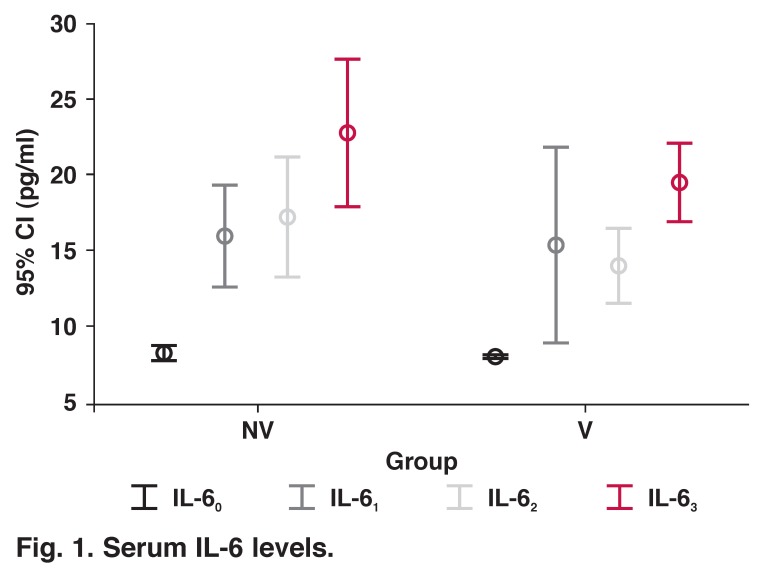
Serum IL-6 levels.

IL-6 levels were increased after induction of anaesthesia in both groups and remained higher than baseline levels six hours after discontinuation of CPB. When the two groups were compared, only IL-6 levels were higher immediately after discontinuation of CPB in the NV group than in the V group (*p* = 0.049) (Table 2, Fig. 1).

**Table 2:** Comparison Of Two Groups With Cytokine Levels And Alveolar–Arterial Oxygen Gradient

*Variable*	*NV group (n = 29) mean ± SD*	*V group (n = 30) mean ± SD*	p-value*
IL-6_0_ (pg/ml)	8.28 ± 1.06	8.03 ± 0.18	0.51
IL-6_1_ (pg/ml)	16.0 ± 9.04	15.37 ± 17.21	0.049
IL-6_2_ (pg/ml)	17.31 ± 10.34	13.97 ± 6.57	0.22
IL-6_3_ (pg/ml)	22.83 ± 12.91	19.50 ± 6.88	0.38
*p*-value**	< 0.001	< 0.001	
IL-8_0_ (pg/ml)	24.86 ± 48.30	25.03 ± 25.53	0.14
IL-8_1_ (pg/ml)	30.79 ± 44.50	26.07 ± 30.35	0.85
IL-8_2_ (pg/ml)	31.72 ± 44.71	21.67 ± 21.18	0.62
IL-8_3_ (pg/ml)	20.34 ± 29.47	24.20 ± 23.63	0.33
*p*-value**	0.005	0.997	
IL-10_0_ (pg/ml)	15.30 ± 7.31	15.58 ± 9.36	0.87
IL-10_1_ (pg/ml)	142.03 ± 55.20	85.74 ± 61.65	0.002
IL-10_2_ (pg/ml)	120.00 ± 41.73	108.45 ± 68.13	0.62
IL-10_3_ (pg/ml)	24.20 ± 24.98	26.16 ± 37.41	0.98
*p*-value**	< 0.001	< 0.001	
Lactate_0_ (mmol/l)	1.56 ± 0.73	1.38 ± 0.47	0.55
Lactate_1_ (mmol/l)	4.07 ± 1.68	2.95 ± 1.49	0.003
Lactate_2_ (mmol/l)	4.10 ± 1.90	4.04 ± 4.04	0.21
Lactate_3_ (mmol/l)	3.96 ± 2.16	4.10 ± 1.37	0.34
*p*-value**	< 0.001	< 0.001	
ΔA–aO_2(0)_ (kPa)	20.60 ± 6.16	11.84 ± 5.39	< 0.001
ΔA–aO_2(1)_ ( kPa)	24.91 ± 3.98	14.15 ± 5.77	< 0.001
ΔA–aO_2(2)_ (kPa)	26.47 ± 5.13	19.54 ± 4.05	< 0.001
ΔA–aO_2(3)_ (kPa)	18.95 ± 7.59	17.19 ± 6.77	0.31
*p*-value**	< 0.001	< 0.001	

SD: standard deviation, NV: non-ventilated, V: ventilated, IL: interleukin, ΔA–aO_2_: alveolar–arterial oxygen gradient. *Mann-Whitney *U*-test, **Friedman test. _0_After induction of anaesthesia, _1_Immediately after discontinuation of CPB, _2_One hour after discontinuation of CPB, _3_Six hours after discontinuation of CPB.

**Fig. 2.**
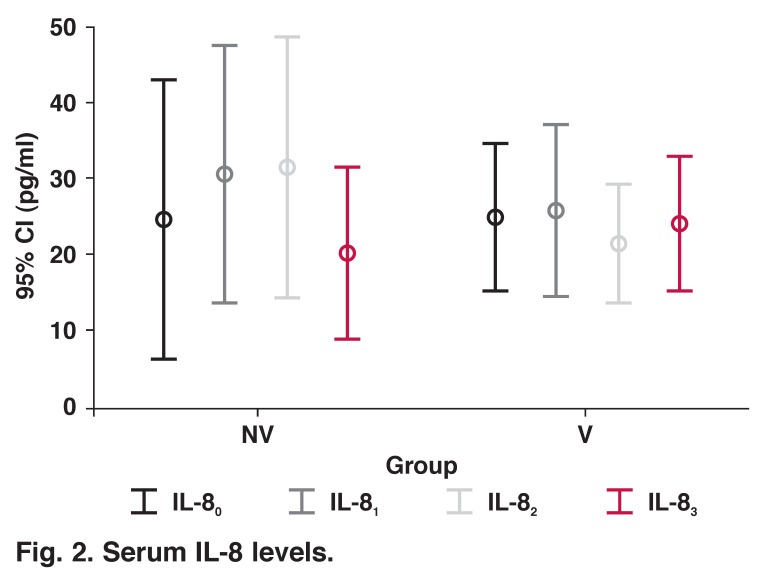
Serum IL-8 levels.

Interleukin-8 levels were increased in the NV group, they peaked one hour after discontinuation of CPB and decreased at six hours to baseline levels (*p* = 0.005). In the V group, IL-8 levels increased, peaked immediately after discontinuation of CPB, decreased at one hour and increased again at six hours, but the change in IL-8 concentrations in this group was not significant (*p* = 0.99). When the two groups were compared, there was no statistically significant difference at any time period (Table 2, Fig. 2).

**Fig. 3.**
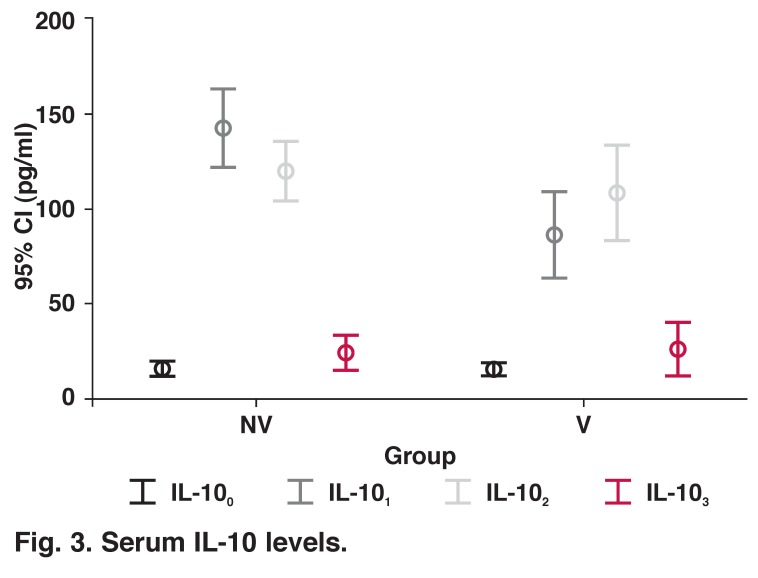
Serum IL-10 levels.

IL-10 levels were increased in the NV group, peaked immediately after discontinuation of CPB, decreased soon after and returned to baseline levels (*p* < 0.001). In the V group, IL-10 levels were increased following induction of anaesthesia, peaked at one hour after discontinuation of CPB, and decreased soon after (*p* < 0.001). When the two groups were compared, only IL-10 levels were higher immediately after discontinuation of CPB in the NV group than in the V group (*p* = 0.001) (Table 2, Fig. 3).

## Pulmonary function

**Fig. 4.**
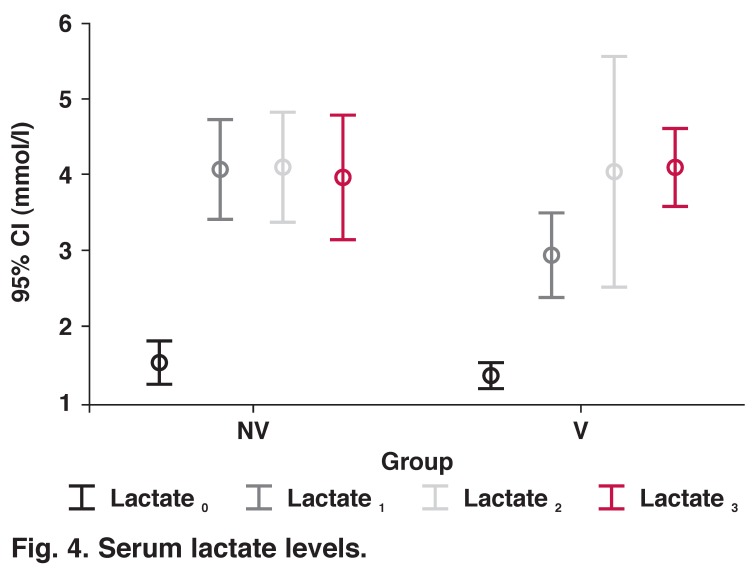
Serum lactate levels.

Lactate release was significantly increased after the induction of anaesthesia in both groups; it peaked at one hour after discontinuation of CPB in the NV group and decreased at six hours, but remained higher than baseline levels (*p* < 0.001). In the ventilated group, levels of lactate progressively increased with time (*p* < 0.001) (Table 2, Fig. 4). However, in each time period there was no significant difference in lactate levels between the groups.

**Fig. 5.**
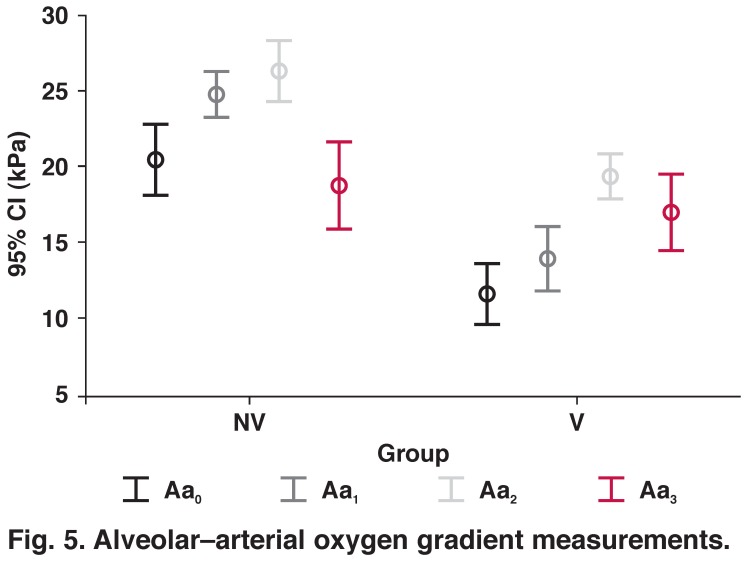
Alveolar–arterial oxygen gradient measurements.

The alveolar–arterial oxygen gradient widened after the induction of anaesthesia, peaked at one hour after discontinuation of CPB, and decreased at six hours, but remained higher than baseline levels in both groups (*p* < 0.001). When the two groups were compared, the gradient was higher in the non-ventilated group after induction, and immediately after and at one hour after discontinuation of CPB (*p* < 0.001) (Table 2, Fig. 5).

In the ICU, the intubation time for the NV group was 9.67 ± 3.29 h and 9.27 ± 2.86 h in the V group (*p* = 0.61) (Table 3). Prolonged intubation was not required in any patient.

**Table 3:** Comparison Of Two Groups By Postoperative Variables

	*NV group (n = 29) mean ± SD*	*V group (n = 30) mean ± SD*	p-value*
ICU intubation time (h)	9.67 ± 3.29	9.27 ± 2.86	0.61
Length of stay			
ICU (h)	47.90 ± 14.16	45.83 ± 2.15	0.43
Postoperative (days)	5.45 ± 0.87	6.07 ± 1.66	0.08
Drainage tubes removed (h)	36.03 ± 9.22	36.93 ± 20.64	0.94
Total amount of drainage (ml)	709.66 ± 541.21	720.00 ± 540.37	0.83
Number of FFP used	1.07 ± 2.18	1.10 ± 1.34	0.94
Number of packed RBC used	1.79 ± 1.67	1.60 ± 1.67	0.66
Number of PC used	0.45 ± 1.32	0.37 ± 1.21	0.80
	n (%)	n (%)	p-value**
Postoperative exploration for haemorrhage	0	1 (3.3)	1.00
Postoperative AF	6 (20.7)	3 (10.0)	0.29
Renal dysfunctiona	5 (17.2)	4 (13.3)	0.73
Postoperative stroke	0	0	-

SD: standard deviation, NV: non-ventilated, V: ventilated, ICU: intensive care unit, FFP: fresh frozen plasma, RBC: red blood cells, PC: platelet concentrate, AF: atrial fibrillation. *Independent samples *t*-test, ** Fisher’s exact test. _a_Defined when peak creatinine value was ≥ 1.5 times the pre-operative value.

## Clinical parameters

Length of stay in ICU and postoperative hospital length of stay were not statistically different in both groups. There was no significant difference when amount of drainage, amount of blood and blood products used, and incidence of postoperative exploration for haemorrhage were compared. Renal failure was defined as peak creatinine value ≥ 1.5 times the pre-operative value and there was no significant difference between the groups (Table 3).

When postoperative AF was studied, there were six patients (20.7%) with AF in the NV group, and three (10.0%) in the V group (*p* = 0.29). Normal sinus rhythm was maintained in all patients except one in the V group. There was no statistically significant difference when maintenance of sinus rhythm was compared. There was no postoperative stroke and mortality noted throughout the study.

## Discussion

The inflammatory response to CPB is acute, complex and can lead to significant morbidity and mortality. It involves the release of cytokines, which may have an impact on postoperative outcomes. The release of cytokines [tumour necrosis factoralpha (TNF-α), interleukins and interferons] may negatively affect cardiac, renal and pulmonary functions.^2^ Cytokine release was shown to correlate with outcome after cardiac surgery. During and after CPB, serum levels of IL-6, IL-8 and TNF-α were increased.

TNF-α is a proximal mediator in the inflammatory cascade.^7^ It induces endothelial cells to produce cell adhesion molecules (Eand P-selectins), which bind to ligands on activated leukocytes to mediate ‘rolling’ of the leukocytes, and intracellular and vascular cell adhesion molecules, which facilitate leukocyte migration from the endothelium to the extravascular space.^1^ TNF-α also induces a second wave of cytokines such as IL-6, which is an important regulator of the hepatic acute-phase response, and IL-8, a cytokine with important neutrophil-activating and chemo-attractant properties.^7^ The neutrophilic effects of IL-8 lead to local release of proteolytic enzymes, with resultant pulmonary injury.^8^

Increased levels of IL-6 and IL-8 were also related to regional wall motion abnormalities following on-pump cardiac surgery.^2^ The release of IL-6 and -8 during CPB led to neutrophil entrapment in pulmonary capillaries. This in turn led to endothelial cell swelling, plasma and protein extravasation into the pulmonary interstitial tissue, together with the release of proteolytic enzymes and the resultant congestion of the alveoli.^9^ The clinical result was decreased P_a_O_2_ and increased P_a_CO_2_, indicating pulmonary injury.^10^

The negative effects of these cytokines are limited by the anti-inflammatory cytokine IL-10, which has protective effects against organ dysfunction following CPB.^11^ In a study of 13 patients, Deblier *et al.*^12^ reported increased IL-10 release, with a peak at the end of CPB in both ventilated and non-ventilated groups, but ventilation did not influence this increase and there was no significant difference between the groups. They failed to document a significant increase in IL-6 in both groups. Beer *et al.*^13^ conducted a similar study and indicated that continued ventilation during CPB attenuated pro-inflammatory IL-6 concentrations and anti-inflammatory IL-10 concentrations. The levels of IL-6 and IL-10 peaked at the end of surgery (which corresponds to T2 in our study) in both groups.

Ng *et al.*^3^ documented higher IL-10 levels one hour after declamping the aorta in the ventilated group (which corresponds to sometime between T_1_ and T_2_ in our study). In our study, the levels of IL-6 were highest six hours after discontinuation of CPB in both groups, but there was no significant difference at any time interval between the ventilated and non-ventilated groups. In the NV group, IL-10 levels peaked immediately after discontinuation of CPB, and one hour after discontinuation of CPB in the V group. The serum IL-10 levels were only significantly higher in the NV group immediately after discontinuation of CPB.

Miranda *et al.*^7^ compared different ventilation strategies during CPB in their randomised, controlled clinical study: continuous ventilation, early and open-lung concepts. They observed that CPB caused a significant increase in IL-6, IL-8 and IL-10 levels. IL-6 levels did not differ significantly between the groups. The decrease in IL-10 levels was more pronounced in the early open-lung group. After discontinuation of CPB, they observed a more rapid decrease in IL-8 in the open-lung groups. They concluded that the open-lung concept attenuates the inflammatory response. It is not wise to compare our results with theirs since the design of the studies were completely different, but we can conclude that we did not observe an objective attenuation of the inflammatory response with ventilation.

Most of the studies comparing the effects of ventilation on the inflammatory response after CPB did not study serum IL-8 levels.^12^,^13^ IL-8 is a very important cytokine in the pathophysiology of myocardial ischaemia. Its levels correlate with complications following myocardial infarction, and anti-IL-8 antibodies prevented injury in experimental models.^7^ Its levels also correlated well with left ventricular wall motion abnormalities in the postoperative period.^4^ Furthermore, it was reported that cyclic alveolar stress due to mechanical ventilation led to increased IL-8 levels.^14^

Ng *et al.*^3^ studied IL-8 levels. They reported IL-8 levels were higher in the non-ventilated group four hours after declamping. In our study, in the ventilated group, we did not observe any significant change in IL-8 concentrations. They peaked at one hour after discontinuation of CPB in the non-ventilated group and then decreased to baseline levels. However, there was no statistically significant difference between the groups at any given time interval.

Lamarche *et al.*^15^ reported that CPB with reperfusion without aortic clamping induced a selective decrease in endothelial relaxation to acetylcholine, and normal ventilation during CPB prevented changes in endothelium-dependent relaxation of the endothelium. Similarly Gagnon *et al.*^4^ documented that continued ventilation during CPB correlated with an attenuated inflammatory and proteolytic process and better preserved pulmonary function. It was suggested that maintaining ventilation and pulmonary flow during CPB attenuated the inflammatory response.^3^,^5^,^16^ The continuation of pulmonary blood flow during CPB also led to reduction in serum IL-6 and -8 levels.^10^

The lungs depend on three separate sources of oxygen delivery: bronchial arterial and pulmonary arterial circulations, and alveolar ventilation.^3^ Two of these cease during CPB because in standard CPB surgery, the lungs are deflated and pulmonary blood flow is shunted. After weaning from CPB, pulmonary reperfusion leads to I/R injury with the release of oxygen free radicals and the resultant lipid peroxidation and endothelial damage.^4^ As a consequence of I/R injury, pulmonary vascular endothelial dysfunction results in secondary vasoconstriction and increased vascular permeability, and finally pulmonary hypertension, oedema and hypoxia.^15^

The alveolar–arterial oxygen gradient is widened and lactate is released as a result of lung injury. The levels of pulmonary lactate release correlate well with systemic lactate levels. This lactate release is increased in procedures employing CPB and correlates with prolonged respiratory support.^6^ Furthermore, deflated/unventilated lungs during CPB induce atelectasis,^12^ which may further induce pro-inflammatory cytokine production.^17^ Atelectasis is also blamed for post-CPB pulmonary injury. Moreover, the degree of intrapulmonary shunt and atelectasis was found to be correlated.

Computed tomography studies also showed a correlation between atelactatic areas and intrapulmonary shunt.^18^ Based on this finding ventilation was suggested to protect lungs from ischaemic damage.

Gasparovic *et al.*^6^ studied pulmonary lactate release and changes in ΔA–aO_2_ during CPB. They reported that the lungs were a significant source of lactate release and there was a correlation between pulmonary lactate release and peripheral lactate concentrations. The duration of CPB correlated with increased pulmonary lactate release and widened ΔA–aO_2_. We also observed that lactate release was increased with CPB and ΔA–aO_2_ was widened in both groups, but unlike their study, we studied the effects of continued ventilation on ΔA–aO_2_ and peripheral lactate levels in order to document its effects on pulmonary functions.

The lactate levels were significantly lower only immediately after discontinuation of CPB in the ventilated group; in other determined time periods there was no difference. In each group, ΔA–aO_2_ widened with time. When the two groups were compared, ΔA–aO_2_ was lower in the ventilated group in all time periods, except for six hours following discontinuation of CPB, when there was no difference. We also report that ΔA–aO_2_ was higher following induction of anesthaesia in the non-ventilated group, and this may be the cause of differences in the following time periods.

In an experimental porcine study, Imura *et al.*^19^ documented that low-frequency ventilation caused reduction of ischaemic changes and lower rates of atelectasis. They also observed that lactate levels were lower in the ventilated group compared to the control. We also documented similar results with regard to ΔA–aO_2_, but lactate levels were higher only in the non-ventilated group immediately after discontinuation of CPB.

Schreiber *et al.*^20^ revealed that to date, there have been three trials that investigated the effects of continued ventilation during CPB on pulmonary functions. Gagnon *et al.*^4^ determined a tidal volume of 3 ml/kg during CPB and John *et al.*^21^ 5 ml/kg. Boldt *et al.*^22^ continued normal ventilation during CPB. They all failed to document a significant difference with regard to ΔA–aO_2_. Only John *et al.*^21^ reported less extravascular lung fluid accumulation and a decreased period of ventilation.

In our study, we employed a modified ventilation strategy, the tidal volume was 5 ml/kg, but frequency was 5/min to avoid an uncomfortable operating field. Different from these studies, we documented higher ΔA–aO_2_ in the non-ventilated group, except for six hours after discontinuation of CPB.

The effects of continued ventilation during CPB on pulmonary function are still controversial. This controversy is very briefly summarised by Schreiber *et al.*^20^ in a recent systematic review and meta-analysis comprising 814 participants of 16 randomised trials. They concluded that continued ventilation was beneficial when serum levels of inflammatory cytokines were considered. Moreover, oxygenation parameters were improved and shunt fraction was decreased. However, all these documented effects were short-lived, questionable, and did not influence the clinical course and final outcomes.

A similar review reported by Vohra *et al.*^23^ also concluded that no convincing results of any ventilation strategies were available. In our study, aside from the cytokine levels and other formula measurements, the clinical outcomes were not affected by continuous ventilation during CPB. The intubation time, length of stay in ICU and hospital, and occurrence of postoperative adverse events were not different between the groups.

## Conclusion

We did not document an objective finding that continuous, low-frequency ventilation attenuated the inflammatory response and positively affected postoperative pulmonary functions, adverse events and outcomes. We believe that further studies with increased numbers of patients and more detailed investigation of inflammatory markers should be done to be able to draw any conclusions.
